# Quality improvement project to improve adherence to lung protective ventilation guidelines

**DOI:** 10.1136/bmjoq-2023-002638

**Published:** 2024-05-24

**Authors:** Adam Harriman, Katrina Butler, Dhruv Parekh, Jonathan Weblin

**Affiliations:** 1 University Hospitals Birmingham NHS Foundation Trust, Birmingham, UK; 2 Birmingham Acute Care Research Group, University of Birmingham Institute of Inflammation and Ageing, Birmingham, UK

**Keywords:** Critical care, Healthcare quality improvement, Quality improvement, Clinical Governance

## Abstract

**Introduction:**

Lung protective ventilation (LPV) is advocated for all patients requiring mechanical ventilation (MV), for any duration of time, to prevent worsening lung injury. Previous studies proved simple interventions can increase awareness of LPV and disease pathophysiology as well as improve adherence to LPV guidelines.

**Objective:**

To assess the impact of a multi-component LPV quality improvement project (QIP) on adherence to LPV guidelines.

**Methods:**

Tidal volume data for all patients requiring MV at a large, tertiary UK critical care unit were collected retrospectively over 3, 6 months, Plan-Do-Study-Act cycles between September 2019 and August 2022. These cycles included the sequential implementation of LPV reports, bedside whiteboards and targeted education led by a multispecialty working group.

**Main outcome measure:**

Adherence against predetermined targets of <5% of MV hours spent at >10 mL/kg predicted body weight (PBW) and >75% of MV hours spent <8 mL/kg PBW for all patients requiring MV.

**Results:**

408 949 hours (17 040 days) of MV data were analysed. Improved LPV adherence was demonstrated throughout the QIP. During mandated MV, time spent >10 mL/kg PBW reduced from 7.65% of MV hours to 4.04% and time spent <8 mL/kg PBW improved from 68.86% of MV hours to 71.87% following the QIP. During spontaneous MV, adherence improved with a reduction in time spent >10 mL/kg PBW from baseline to completion (13.2% vs 6.75%) with increased time spent <8 mL/kg PBW (62.74% vs 72.25%). Despite demonstrating improvements in adherence, we were unable to achieve success in all our predetermined targets.

**Conclusion:**

This multicomponent intervention including the use of LPV reports, bedside whiteboards and education improves adherence to LPV guidelines. More robust data analysis of reasons for non-adherence to our predetermined targets is required to guide future interventions that may allow further improvement in adherence to LPV guidelines.

**WHAT IS ALREADY KNOWN ON THIS TOPIC**

- It is widely acknowledged that lung protective ventilation reduces morbidity and mortality in patients requiring mechanical ventilation. Adherence in clinical practice can be challenging due to human and non-human factors.

**WHAT THIS STUDY ADDS**

- This single-centre, quality improvement study highlights simple practical strategies that can improve adherence to lung protective ventilation guidelines. Adherence is more challenging during spontaneous modes of ventilation.

**HOW THIS STUDY MIGHT AFFECT RESEARCH, PRACTICE OR POLICY**

- The study may guide local standard practice policy and key performance indicators with regard to lung protective ventilation but highlights the need for continual and robust understanding of local or individual reasons for non-adherence.

## Introduction

Lung protective ventilation (LPV) has become a corner stone of the management of patients admitted to critical care with acute lung injury, requiring mechanical ventilation (MV) since the outcome of the Acute Respiratory Distress Syndrome Network (ARDSNET) trial in 2000.^1^ Patients diagnosed with acute respiratory distress syndrome (ARDS) managed with restricted tidal volumes (Vt) of 6–8 mL/kg predicted body weight (PBW) had a lower morbidity and mortality compared with those in the control group ventilated at 10–15 mL/kg PBW.

There is now increasing evidence supporting the benefits of restricting Vt in non-ARDS, mechanically ventilated populations.^2^ Futier *et al* highlighted that LPV strategies implemented during the intraoperative period reduced the risk of postoperative pulmonary complications, as well as those only requiring short periods of MV.^3^ Serpa Neto *et al* also found a positive correlation between the use of low Vt ventilation and clinical outcomes in patients without ARDS.^4^ The use of restricted Vt for all patients requiring MV has subsequently become common practice.^5^

Since the initial conception of LPV, research focus has shifted towards the implementation of LPV guidelines, including strategies to optimise adherence and address potential barriers to poor compliance. Strategic implementation of LPV guidelines in patients with ARDS has been shown to be reduced as a direct consequence of under-recognition of the disease in clinical practice.^6^ Numerous supplementary trials have presented reasons for the lack of implementation of LPV.^7–9^ Under-recognition of ARDS remains the most frequently reported factor, with a lack clear protocols, poor education surrounding pathology/management, concerns regarding patient hypoventilation and inadequate staffing to complete labour-intensive management, all being reported as limiters to implementation.

To increase adherence to LPV guidelines, interventions, such as audit, feedback and education of clinicians, have been used successfully.^10 11^ The development of protocols in the form of displayed written LPV targets has shown promise in improving compliance with LPV in the short term and long term in multiple large trials.^12–14^ The creation of a specialist ‘LPV team’ either from single or multi specialties has also proved effective in increasing awareness, diagnosis and the management of patients with ARDS.^15 16^ Finally, modern electronic surveillance techniques including the use of a clinical decision support tool (CDS) can be used to identify ARDS and alert clinicians, improving recognition.^9 17^ Recently, Short *et al* have demonstrated improved compliance with LPV strategies by up to 16% through the implementation of a CDS tool which monitored electronic records and suggested changes to ventilator parameters.^18^ While these authors have presented interventions to improve adherence to local LPV guidelines, often these are used as single interventions and not as part of a comprehensive bundle.

The COVID-19 pandemic re-enforced the importance of LPV in clinical practice in the Intensive Care Unit (ICU) due to the disease pathology^19^ and highlighted shortcomings within the practice across our large multispecialty critical care. Subsequently, a working group was established to look at initiatives to improve local LPV adherence.

## Methods

A preliminary retrospective audit was undertaken to review and analyse the delivered Vt of all mechanically ventilated patients admitted to a large, 100-bed, UK tertiary ICU between September 2019 and February 2020. The critical care under analysis encompasses four different clinical specialties, including cardiothoracic, burns and trauma, neurology, neurosurgery and specialist surgery.

For patients admitted to ICU, as part of standard nursing care, hourly patient observations including ventilation parameters and Vt’s are inputted onto an electronic prescribing, information and communication system (PICS). These data are automatically collated into a health informatics database which generates a report stratifying delivered Vts into four risk categories based on patients most recently documented height.

1. <6 mL/kg PBW.
2. 6–8 mL/kg PBW.
3. 8–10 mL/kg PBW.
4. >10 mL/kg PBW.

Based on evidence-based practice, local guidelines mandate patients should be ventilated at a target <8 mL/kg PBW.^20^

### COVID-19 pandemic

Between March and May 2020, the repurposing of the multidisciplinary team (MDT) in response to the COVID-19 pandemic led to the introduction of a physiotherapy-led ward round to support adherence to LPV guidelines for patients requiring MV. This included calculation of target ranges for LPV Vt based on 6–8 mL/kg PBW, alongside twice daily ventilation ward rounds, to ensure adherence or make the necessary adjustments to maintain LPV. Following the pandemic and the restoration of normal National Health Services (NHS), the ventilation ward round was disbanded.

**Figure 1:**
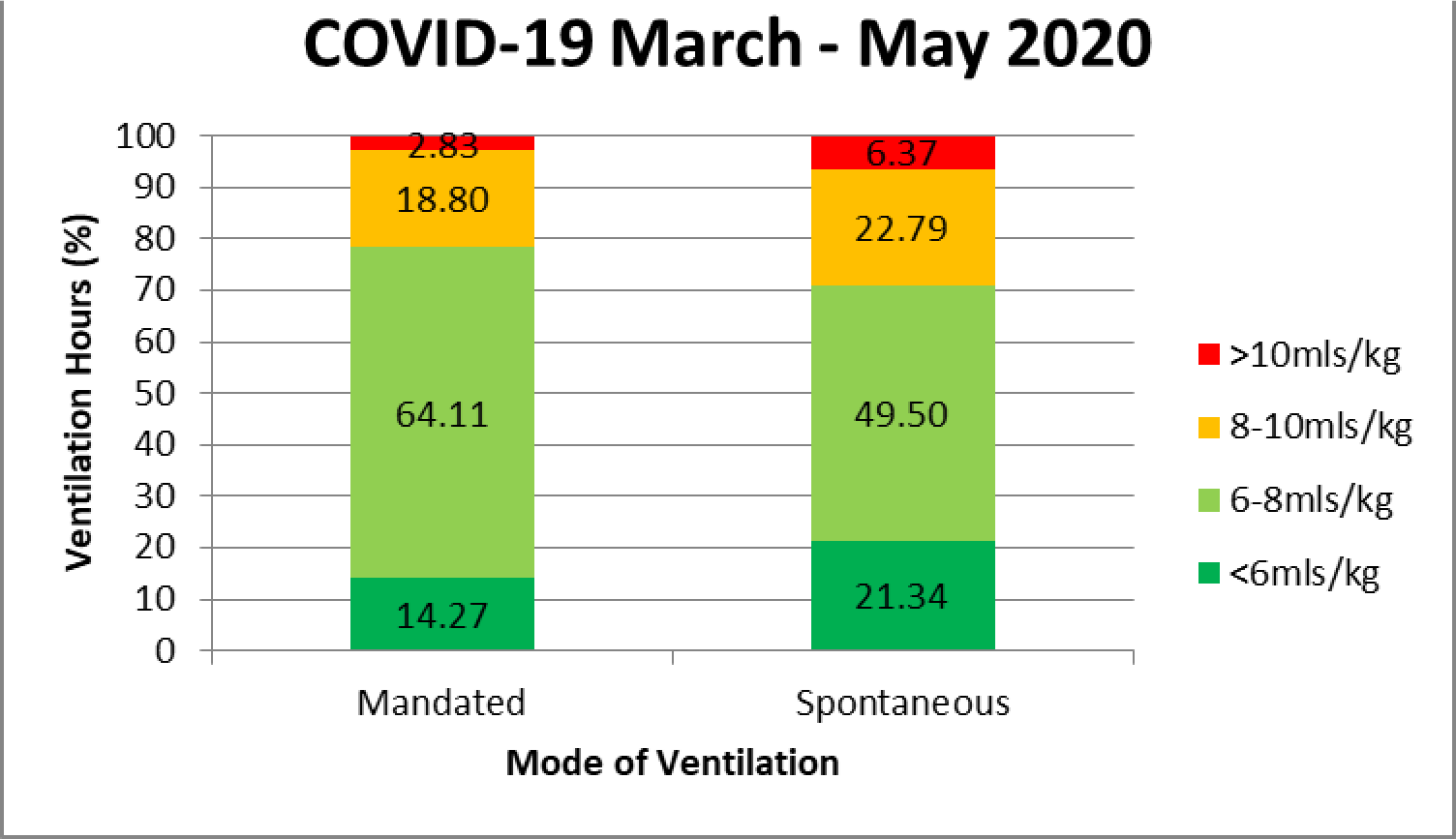
Adherence to LPV targets during COVID-19 pandemic. LPV, lung protective ventilation.

Based on the evaluation of LPV compliance with both mandated (n=36 350 hours of MV) and spontaneous (n=17 372 hours of MV) ventilation modes, against evidence-based practice during this period (figure 1), an expert panel of critical care consultants and physiotherapists came to a consensus on the following local LPV targets for all patients requiring MV.

- Target 1: Achieve <5% time spent mechanically ventilated at >10 mL/kg PBW.
- Target 2: Achieve >75% of the time mechanically ventilated at <8 mL/kg PBW.

While these agreed targets were below the level of compliance achieved during the pandemic, it was felt adherence was amplified by the homogeneous nature of COVID-19 patients and enhanced focus on LPV as the primary treatment method, in addition to the ventilation ward rounds. As a multispecialty ICU, it was felt these targets were realistic given specific cohorts may require ventilation outside LPV that is, neuroprotective ventilation measures.

### Design

In February 2021, an MDT workforce group including medical, nursing and physiotherapy staff, was formed with the sole purpose of championing LPV and developing strategies to improve adherence to local LPV guidelines across all specialties. Three, 6 months Plan-Do-Study-Act (PDSA) cycles were planned as part of the quality improvement project (QIP). MV data from PICS were collected prospectively by the hospital’s health informatics team with interim analysis of LPV adherence completed after each stage of the PDSA cycle.

The project was deemed a service evaluation and registered with the local CARMS team (Code CARMS-14090).

### Strategy

#### PDSA cycle 1 (March–August 2021)

**Figure 2:**
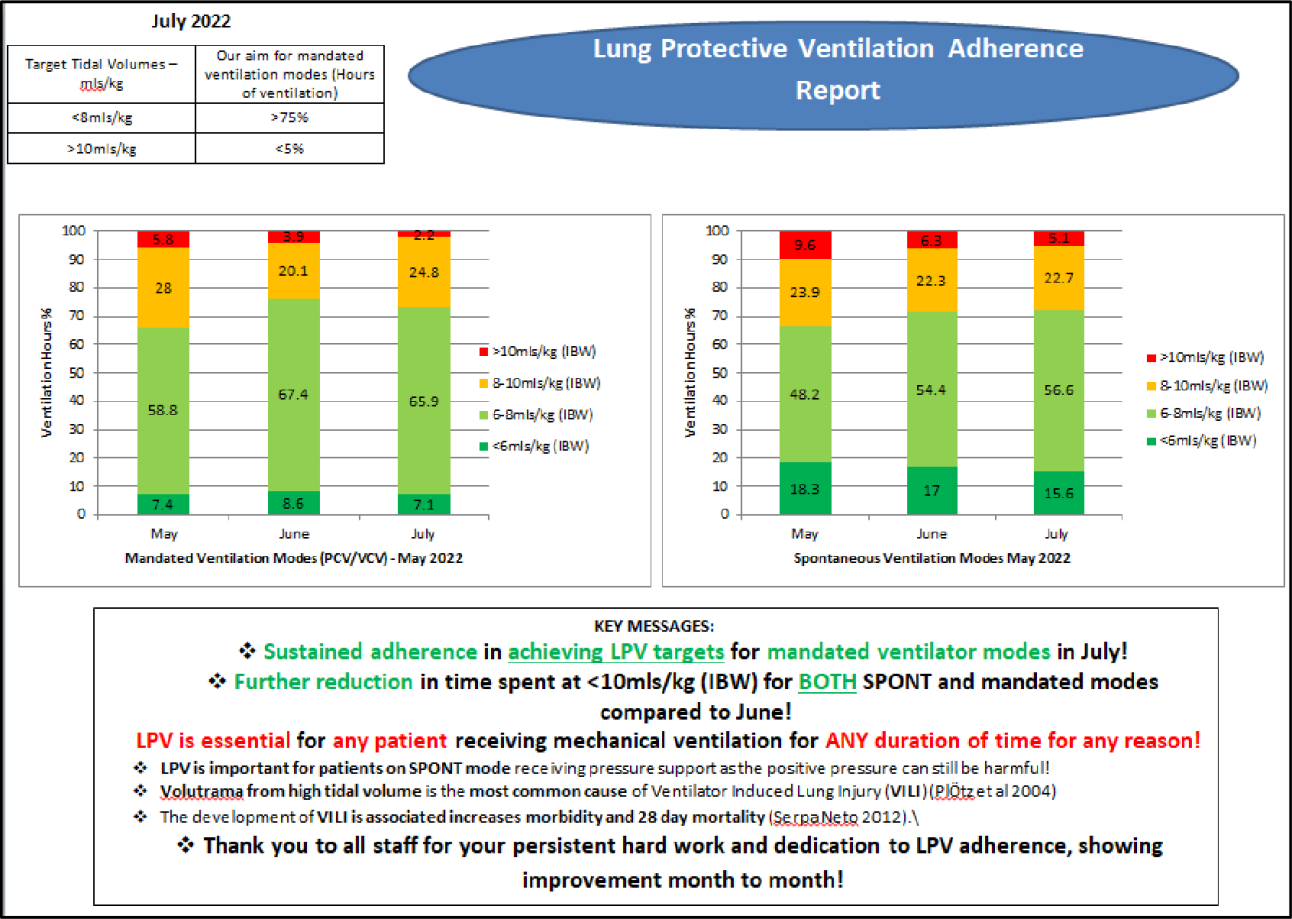
Example of a monthly LPV adherence report. LPV, lung protective ventilation. IBW; Ideal Body Weight, PCV; Pressure Control Ventilation, VCV; Volume Control Ventilation.

The results of the baseline audit were presented to consultants and senior critical care clinicians in a clinical specialty and governance meeting to highlight the shortcoming of the current service and the need for improvement. The implementation of monthly LPV adherence reports displaying monthly, and the preceding 2 months, performance against the predetermined local targets and ‘key messages’ regarding the importance of LPV adherence to patient outcomes were disseminated among senior clinicians and displayed in clinical areas to improve awareness and compliance with LPV (figure 2). LPV guidelines were made accessible to the MDT in electronic end of the bed folders and laminated cards with IBW calculated using 6–8 mL/kg for a range of heights and were attached to the ventilator in the bed space as a point of reference for staff.

#### PDSA cycle 2: September–February 2022

A 1-week, snapshot audit, for patients admitted to the ICU requiring MV identified a large number of patient heights inputted onto PICS were estimated, rather than measured. This contributed to inaccurate calculation of patient-specific LPV targets and subsequent overventilation/underventilation. An initiative was implemented to ensure all patients admitted to the ICU requiring MV had their heights accurately measured by the MDT involved in the patient’s care, at the time of ICU admission.

Although patient height/weight and LPV targets are documented in the patient’s electronic patient noting, it was felt LPV targets may have also been lost in the plethora of clinical noting. To improve visibility and awareness of LPV targets, patient’s height and calculated LPV targets were additionally displayed on bedside whiteboards. Fundamentally, this initiative aimed to improve the speed at which clinicians could access the patient’s specific LPV targets at the bedside, as well as provide a visual reminder regarding LPV for all clinicians reviewing the patient.

At this point, the LPV working group felt it was appropriate to stratify adherence to LPV by mandated and spontaneous modes of MV. This was implemented following ad hoc monthly analysis of LPV data which demonstrated a trend towards greater adherence in mandated modes of MV. It was felt adherence to LPV in mandated modes was more clinically achievable due to the challenges in the regulation of VT and work of breathing in spontaneously breathing patients, for example, patients achieving high VT ventilation on invasive continuous positive airway pressure only, and as a result, this guided MDT teaching towards the importance of continuing LPV during spontaneous modes of MV during stage 3 of the QIP.

#### Stage 3: March–August 2022

To supplement monthly audit reports and the displaying of patient-specific LPV targets on bedside whiteboard, the LPV team completed weekly, ad hoc, bedside teaching to the MDT as part of educational ward rounds. The training aimed to improve the awareness and knowledge of the benefits of LPV adherence in all clinical formats but also to empower staff to challenge non-adherence, and where appropriate make changes to the ventilation parameters. MDT ‘LPV champions’ were instated in each critical care specialty to drive LPV adherence on the floor and create a level of accountability.

LPV training was also formally delivered as part of nursing/Allied Health Professionals (AHP)/medical new starter training, providing an overview of LPV literature. Risks of volutrauma, atalectrauma, barotrauma and biotruama with under and over ventilation and the associated morbidity and mortality were covered. Emphasis was placed on LPV being paramount regardless of mode of ventilation or primary diagnosis, and that all MDT members are accountable for its implementation and adherence.

## Results

During all three phases of the QIP, data on 408 949 hours (17 040 days) of MV were analysed. The total numbers of MV hours during each QIP stage are displayed in table 1.

**Table 1:** Total number of MV hours and percentage of time spent at target tidal volumes, during each PDSA cycle

	Target 1: Achieve <5% time spent mechanically ventilated at >10 mL/kg PBW		Target 2: Achieve >75% of the time ventilated at <8 mL/kg PBW	
	Mandated ventilation (hours)	Spontaneous ventilation (hours)	Mandated ventilation (hours)	Spontaneous ventilation (hours)
Pre- QIP	7.65% (3690)	13.2% (4758)	68.86% (33 214)	62.74% (22 617)
PDSA 1	5.16% (2487)	10.34% (3792)	70.26% (33 865)	66.3% (24 314)
PDSA 2	4.48% (2435)	10.01% (3811)	69.85% (37 969)	64.61% (24 596)
PDSA 3	4.04% (1946)	6.75% (3069)	71.87% (34 626)	72.25% (32 850)

MV, mechanical ventilation; PBW, predicted body weight; PDSA, plan-do-study-act; QIP, quality improvement project.

### Target 1: achieve <5% time spent mechanically ventilated at >10 mL/kg PBW

**Figure 3:**
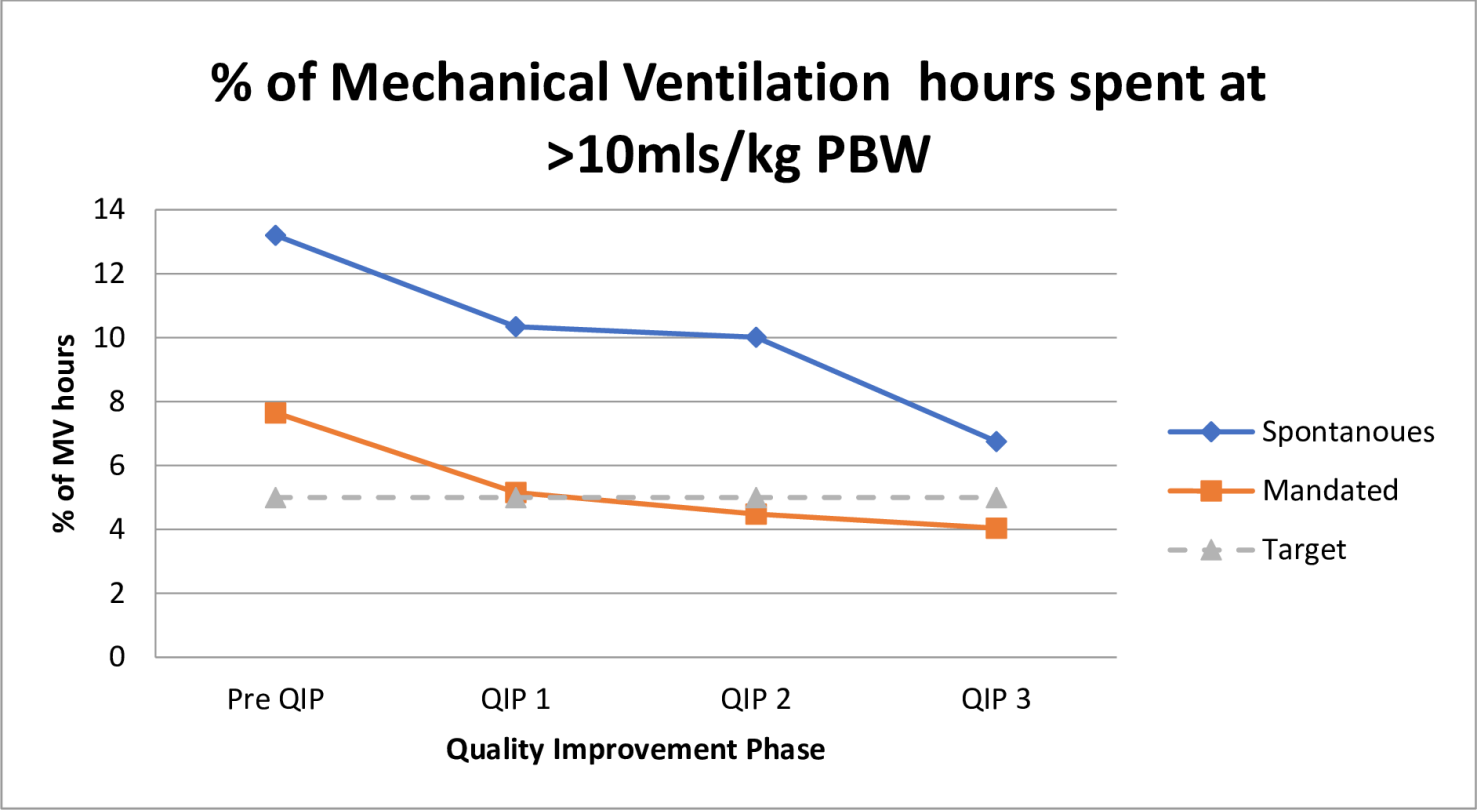
Adherence against target 1. PBW, predicted body weight; QIP, quality improvement project.

As shown in figure 3, pre-QIP, 7.65% (n=3690 hours) of mandated MV hours and 13.2% (4,758 hours) of spontaneous ventilation were spent above 10 mL/kg PBW. Following the first PDSA cycle, 5.16% (2487 hours) of mandated MV hours and 10.34% (3792 hours) of spontaneous ventilation hours were spent above 10 mL/kg PBW. Following PDSA cycle 2, 4.48% (2435 hours) of mandated MV hours and 10.01% (3811 hours) of spontaneous ventilation hours were spent at >10mL/kg PBW. Following PDSA cycle 3, 4.04% (1946 hours) of mandated ventilation hours and 6.75% (3069 hours) of spontaneous ventilation hours were spent >10 mL/kg PBW.

### Target 2: achieve >75% of the time ventilated at <8 mL/kg PBW

**Figure 4:**
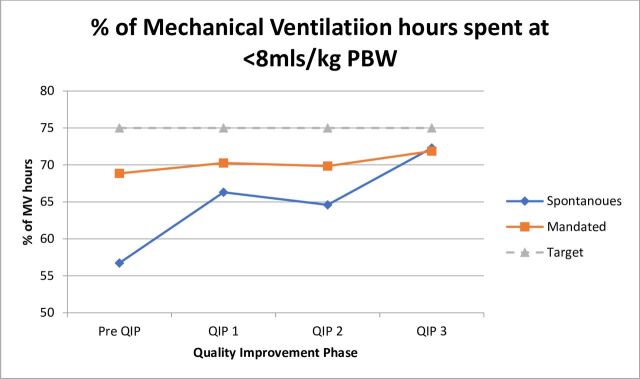
Adherence against target 2. PBW, predicted body weight; QIP, quality improvement project.

As shown in figure 4, pre-QIP, 68.86% (33 214 hours) of mandated MV hours and 62.74% (22 617 hours) of spontaneous ventilation were spent below 8 mL/kg PBW. Following PDSA cycle 1, 70.26% (33 865 hours) and 66.3% (24 314 hours) of mandated and spontaneous MV hours were spent below 8 mL/kg PBW. Following PDSA cycle 2, 69.85% (37 969 hours) and 64.61% (24 596 hours) were spent <8 mL/kg PBW and following PDSA cycle 3, 71.87% (34 626 hours) and 72.25% (32 850 hours), respectively, were spent <8 mL/kg PBW.

## Discussion

This paper reports the multistage QIP initiated at a large, UK, multispecialty critical care unit. The initiative focused on a sequential introduction of targeted interventions to improve adherence with local, evidence-based, LPV guidelines through the use of disseminated monthly LPV adherence reports, bedside whiteboards and MDT education.

### Mandated ventilation modes

Each PDSA cycle was associated with an improvement in adherence to target 1 for mandated modes of MV, with a reduction in the number of MV hours delivered > 10 mL/kg PBW. During phases 2 and 3 of the QIP, less than 5% of MV hours were spent >10 mL/kg PBW, achieving this predetermined target.

There was a trend towards a reduction in the percentage of hours delivered <8 mL/kg PBW during the QIP (68.86% vs 71.87%). We were unable to achieve our predetermined target 2 of <75%, but this still represents a valuable improvement in adherence and reduction in potential patient harm. The ability of our study to interrogate the data and establish reasons for non-compliance to target 2 is limited by our data capture system. Although our electronic database allowed for the analysis of a substantial number of MV hours, it does not allow for patient-specific or case-specific interpretation. This meant that we were unable to comment on individual reasons for poor compliance with LPV targets. Like Knighton *et al*, we can hypothesise that despite our interventions; there may remain elements of a lack of concordance with clinician perception of the requirement of LPV or uncertainty with restricting Vts in certain subsets of patient’s, that is, those requiring neuroprotective ventilation.^9^ However, the improvements seen in adherence to our interventions are consistent with previous studies and published literature on the subject.^11^

### Spontaneous MV modes

We achieved improved adherence to target one during spontaneous ventilation modes, with a reduction in the number of MV hours >10mL/kg PBW from baseline (4758 hours, 13.2%) to QIP completion (3069 hours, 6.75%). There was a trend towards more time spent mechanically ventilated at <8 mL/kg PBW, however, there was some reversibility from phase 1 to 2 of the QIP. However, we did not see any significant cyclical reversibility in our improvements that may be associated with the high turnover of staff within our institution or inexperience with new or junior workforces. We feel that the effect was mitigated through the targeted, interdisciplinary new starter education specifically in stage 3 which now runs at regular intervals during the year. This allowed constant re-enforcement of each intervention as described to be regularly and equally disseminated across the units, creating a culture of focus on LPV. During phase 3 of the QIP, the education package became focused on the importance of LPV during spontaneous MV and the harmful effects of non-adherence to LPV for postsurgical and non-ARDS populations.^3 4^ This targeted education may account for the improvement observed during stage 3. However, we were unable to achieve adherence to both our predetermined targets during spontaneous MV. Given the performance during the QIP, it may be relevant to review our expectations and targets across mandated and spontaneous MV modes in the future as it may be significantly more realistic to achieve a higher degree of LPV during mandated modes, than spontaneous. This decision would need to be supported by a more rigorous analysis of our MV data.

Several human factors may exist for why we were unable to achieve both of our predetermined targets for spontaneous modes of ventilation. First, we identified that while clinicians appreciated the necessity of LPV during mandated ventilation modes, there was less of a consensus among clinical teams regarding the requirement of LPV during spontaneous ventilation. Second, it is possible that some of the hours spent greater than 10 mL/kg PBW during spontaneous modes of MV were delivered on invasive CPAP, and therefore, could not be reduced to within protective ranges through ventilator adjustment alone. However, as later described in detail, the ability to interpret specific instances of poor adherence is limited by our methodology.

## Limitations

A strength of our QIP is the ability of our PICS and health informatics databases to collect vast volumes of MV delivery data. This electronic database allowed robust collection of real-time data, facilitating in-depth analysis and comparison across monthly and yearly basis. However, the database amalgamates ventilation hour’s data into critical care unit or clinical specialty categories only. Specifically, this means that individual patient data and individual instances of adherence could only be analysed by hand. Given the substantial volume of MV hours being dealt with, this was beyond the scope of our methodology and does; therefore, limit our ability to interpret individual instances of non-adherence. Further work in future stages of the QIP could be focused on the reasons for individual instances of poor adherence to set targets and therefore, allow the conception of additional interventions.

## Conclusions

Our experience and presentation of this QIP demonstrate how a multistage, interventional process can improve local adherence to LPV guidelines, thus leading to the enhancement in care provided to patients requiring MV. We believe that our simple intervention methods are transferable to other critical care units of varying sizes and specialty. Future initiatives to further enhance and maintain the culture of LPV across the unit could involve making LPV a specific trust key performance indicator, or the cost–patient benefit of the implementation of a specific LPV ward round, like that implemented at our Trust during the COVID-19 pandemic. Focus on the reasons for poor individual compliance may allow identification of other barriers to implementing LPV at our Trust which may improve LPV adherence and should be a key focus for further cycles of our project.

## Data Availability

All data relevant to the study are included in the article or uploaded as online supplemental information.
